# HMOX1 pathway signature predicts clinical benefit from immunotherapy plus tyrosine kinase inhibitor therapy in advanced renal cell carcinoma

**DOI:** 10.1002/cam4.5787

**Published:** 2023-04-09

**Authors:** Xianglai Xu, Sihong Zhang, Ying Wang, Yanjun Zhu, Jiajun Wang, Jianming Guo

**Affiliations:** ^1^ Department of Urology, Zhongshan Hospital Fudan University Shanghai China; ^2^ Department of Critical Care Medicine, Zhongshan Hospital Fudan University Shanghai China

**Keywords:** heme oxygenase 1, immune checkpoint inhibitor, renal cell carcinoma, T‐cell dysfunction, tyrosine kinase inhibitor

## Abstract

**Background:**

Immunotherapy (IO) plus tyrosine kinase inhibitor (TKI) emerged as standard first‐line therapy for advanced renal cell carcinoma (RCC). The heme Oxygenase 1 (HMOX1) pathway is involved in tumor development and treatment resistance, which may affect the efficacy of TKI + IO.

**Methods:**

Two cohorts from our center (ZS‐MRCC, ZS‐HRRCC), one cohort from clinical trial (JAVELIN Renal 101) and the Cancer Genome Atlas (TCGA‐KIRC) were enrolled. HMOX1 pathway signatures were determined for each sample by RNA‐sequencing and gene set enrichment analysis. Immune infiltration was evaluated by flow cytometry. Response and progression‐free survival (PFS) were set as primary endpoints.

**Results:**

Patients of low‐HMOX1 signature showed higher objective response rate (43.5% vs. 27.3%) in ZS‐MRCC cohort and longer PFS in both cohorts (ZS‐MRCC cohort, *p* = 0.019; JAVELIN‐101 cohort, *p* = 0.036). Patients in the high‐HMOX1 signature arm also showed greater clinical benefit from TKI + IO, rather than TKI monotherapy (*p* < 0.001). In high‐HMOX1 signature RCC tissues, CD8^+^ T cells showed a dysfunctional phenotype with decreased GZMB expression (Spearman's ρ = −0.32, *p* = 0.045). A risk score based on HMOX1 signature was further constructed by random forest approach, involving HMOX1 signature and immunologic features. In patients with a low risk level, TKI + IO combination therapy demonstrated longer PFS than TKI monotherapy (*p* < 0.001), however in individuals with a high risk score group, these two regimens did not give different advantages.

**Conclusions:**

Our study identified the HMOX1 pathway signature was a potential prognostic factor of progression‐free survival for TKI + IO combination therapy in the advanced RCC in different cohort, especially in first‐line management of mRCC in the Javelin 101 cohort. Moreover, HMOX1 signature was associated with T‐cell function in tumor environment.

## Graphical abstract

Our study identified the HMOX1 pathway signature was a potential prognostic factor of progression‐free survival for TKI + IO combination therapy in the advanced RCC in different cohort. Moreover, HMOX1 signature was associated with T‐cell function in TME. The random forest model based on HMOXI signature may improve decision‐making between TKI + IO or TKI monotherapy.

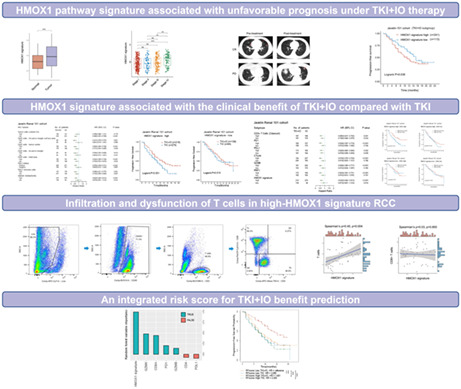

## 1 INTRODUCTION

Renal cell carcinoma (RCC) accounts approximately for 3% of all adult malignancies.^1^ Despite all the improvements made in the past 15 years, metastatic renal cell carcinoma (mRCC) remains incurable, with poor 5‐year survival rates. The management of mRCC has changed tremendously over the past decades. From a severely fatal disease with few therapeutic options beyond cytoreductive nephrectomy, systemic therapy for mRCC today consists of several effective therapy strategies, such as inhibition of the VEGF signaling pathway using VEGFR tyrosine‐ kinase inhibitors (VEGFR TKIs) or anti‐VEGF‐A antibody, inhibition of the mTOR signaling pathway and immune‐checkpoint inhibitors (ICIs).^2^ In recent years, clinical trials of ICI‐based combination therapy (containing a VEGFR TKI) have demonstrated extraordinary success in patients with mRCC.^3^, ^4^, ^5^ As a result, the European Association of Urology (EAU) Guideline of RCC recommends three TKI plus immunotherapy (TKI + IO) combinations, including axitinib plus pembrolizumab, cabozantinib plus nivolumab, and lenvatinib plus pembrolizumab as standard‐of‐care first‐line therapy for treatment‐naive mRCC.^6^

Although TKI + IO therapies have dramatically improved the prognosis for patients with mRCC, the incidence of serious side events has increased, which potentially requires treatment discontinuation. Thus, biomarkers that predict TKI + IO therapy benefits are essential for identifying the optimal therapy group. With proper biomarkers, it is feasible to improve the response rate of patients from TKI + IO treatment, limiting the exposition to ineffective medication and avoiding potential side events. However, biomarkers for ICI monotherapy, including intratumoral PD‐L1 expression, single gene mutations, tumor mutational burden, or frameshift indel load, have been demonstrated to be unreliable for predicting the TKI + IO benefits of mRCC.^7^, ^8^, ^9^ Our study aims to collect multi‐omics data and develop a predictive factor of response to immunotherapy‐based combinations in mRCC.

The heme oxygenase (HO) family of enzymes is the key‐limiting enzyme in the degradation of heme and production of carbon monoxide (CO), ferrous iron (Fe2+), and biliverdin products.^10^ There are two isoforms in human beings: HO‐1, which is induced in response to external stimuli, such as oxidative stress and cytokines; HO‐2, which is constitutively expressed.^11^, ^12^ At the transcript level, HO‐1 is encoded by HMOX1, while HO‐2 is encoded by HMOX2. At the cellular level, HO‐1 has most frequently been reported to be expressed by tumor cells and tumor‐associated macrophages (TAMs).^13^ The HO‐1 isoform has been shown to be expressed in a wide spectrum of malignancies, including renal carcinoma,^14^ and implicated in a vast array of biological processes which may promote tumor progression and metastasis.^13^, ^15^, ^16^, ^17^, ^18^ These biological functions of HMOX1 pathway may be attributed to CO, which coordinates multiple signaling pathways including p38 MAPK, STAT1/3, and NF‐κB, which is essential for CD8^+^ T‐cell effector function.^19^, ^20^, ^21^, ^22^ Consequently, HMOX1 pathway activation may hinder antitumor CD8^+^ T‐cell function in the tumor microenvironment (TME).

In the present study, we constructed an HMOX1 pathway signature describing HMOX1 pathway activation status based on RNA‐sequencing data in four independent RCC cohorts. The HMOX1 pathway signature was assessed as a novel potential predictive biomarker for TKI + IO therapy.

## 2 MATERIALS AND METHODS

### 2.1 Study cohorts and data collection

Current study comprised participants from four independent RCC cohorts. We initially analyzed the HMOX1 signature expression in the TCGA cohort and demonstrated that HMOX1 signature expression was elevated in RCC tissues and correlated with tumor stage and grade. The Cancer Genome Atlas (TCGA) project included 530 clear cell RCC patients in the TCGA‐KIRC cohort (https://xena.ucsc.edu/).^23^ Clinical, pathologic, RNA‐seq, somatic mutation, and follow‐up data were also downloaded from the UCSC xena browser.

Second, the HMOX1 signature was evaluated in the ZS‐MRCC cohort and was found to be a TKI + IO prognostic signature. Fifty‐one metastatic RCC patients were enrolled in the ZS‐MRCC cohort at Zhongshan Hospital, Fudan University, and treated with TKI + IO combination therapy between January 2017 and December 2020. Detailed inclusion and exclusion criteria are listed in Table S1. Inclusion criteria for patients were a diagnosis of metastatic RCC, treatment with a combination of TKI and IO, absence of other malignancy, and availability of tumor tissue. After excluding six patients due to the unavailability of tissue samples or loss of follow‐up, 45 patients matched the inclusion criteria. Clinical, pathologic information, treatment response, and survival were retrospectively obtained from medical records. The RECIST 1.1 criteria were utilized to define therapeutic response and disease progression.^24^ Table S2 shows the baseline demographic and clinical characteristics of the ZS‐MRCC cohort.

To confirm the prognostic value of HMOX1 signature, the JAVELIN Renal 101 (JAVELIN‐101) cohort was used, which enrolled 726 metastatic RCC patients with advanced RCC, treated by TKI + IO (*n* = 354) or TKI monotherapy (*n* = 372), in a phase III clinical trial of TKI + IO (avelumab+axitinib) versus TKI monotherapy (sunitinib).^3^ Inclusion and exclusion criteria were described in a previous study.^3^ Clinical, pathologic, RNA‐seq, somatic mutation, and follow‐up data were acquired from previous studies by Motzer et al.^3^, ^25^

Finally, we utilized the ZS‐HRRCC cohort to evaluate the connections between HMOX1 signature and TME, specifically T cells, CD8^+^ T cells, and CD8^+^ T‐cell function. The ZS‐HRRCC cohort included 43 patients with high‐risk localized RCC who had undergone radical nephrectomy at Zhongshan Hospital, Fudan University from January 2020 to December 2021. Detailed inclusion and exclusion criteria are listed in Table S1. Inclusion criteria included surgically resected localized or locally progressed RCC, a high risk of recurrence, the absence of neoadjuvant therapy, and the availability of tumor samples. After removing three patients due to the unavailability of tissue samples or failure to meet sample quality control standards, 40 patients met the inclusion criteria. Clinical and pathologic information was retrospectively obtained from medical records.

### 2.2 RNA‐seq and data processing

The MagBeads Total RNA Extraction Kit (MAJORIVD) was used to isolate total RNA by the manufacturer's instructions. RNA was further purified with the RNAClean XP Kit (Beckman Coulter) and RNase‐Free DNase Set (QIAGEN). Library construction and sequencing were performed by Shanghai Biotechnology Corp. (Shanghai, China). RNA libraries were prepared with the VAHTS Universal V6 RNA‐seq Library Prep Kit for Illumina (Vazyme) and sequenced utilizing the NovaSeq 6000 equipment (Illumina). Sequencing data were finally normalized to both FPKM and read count values.

### 2.3 Flow cytometry

Before surgery, peripheral blood samples were collected from venous blood and preserved in heparin anticoagulant tubes at 4°C until experimentation (within 2 h). White blood cells were extracted by RBC Lysis Buffer (Thermo Fisher Scientific). RCC samples were obtained and examined just after surgical resection. At 37°C, freshly minced tumor samples were digested with collagenase IV (Sigma) and DNase I (Sigma), and then strained through a 70 μm strainer. The samples were then treated in RBC lysis buffer (Thermo Fisher Scientific). After blocking Fc receptors, single‐cell suspensions and white blood cells were stained separately for 30 min at 4°C with fluorescently labeled membrane marker antibodies. Intracellular proteins were stained with appropriate antibodies after being dissolved in Intracellular Fixation & Permeabilization Buffer (Thermo Fisher Scientific) according to the manufacturer's instructions. White blood cells and cell suspensions were stained with antibodies labeled with fluorochrome and maintained with cell staining buffer. Flowjo v10.0 was used to analyze BD LSRFortessaTM X‐20 (BD Biosciences) FACS data (Tree Star). Antibodies in detail are described in a previous publication.^26^

### 2.4 In silico approaches

Single sample gene set enrichment analysis (ssGSEA) was also performed using the “GSVA” R package to get sample‐level scores for HMOX1 pathway signature.^27^ The HMOX1 pathway signature genes were obtained from the REACTOME REGULATION OF HMOX1 EXPRESSION AND ACTIVITY pathway in the REACTOME dataset, as specified in MSigDB^28^ (Table S3). COX and Kaplan–Meier analyses were performed by the “survival” and “survminer” R packages. Then, the cutoff of high versus low was calculated by the “survminer” R packages, and set at 32%. Those whose HMOX1 signature expression was higher than the cutoff belonged to the high‐HMOX1 subgroup. The cutoff was the same for both the ZS‐MRCC cohort and the Javelin‐101 cohort. The HMOX1 signature genes were same across all cohorts. The Forest plots were plotted by the “forestplot” R package. The waterfall plot was calculated and plotted by the “ComplexHeatmap” and “ggplot2” R packages. Finally, random forest model construction was obtained by the “randomForestSRC” and “ggRandomForests” R packages. All analysis approaches were performed on the platform of R software (https://www.r‐project.org/).

### 2.5 Statistical analysis

The categorical variables were evaluated using the chi‐square test, Fisher's exact test, or the Cochran–Mantel–Haenszel test, as applicable. The Wilcoxon signed ranks test was used to compare continuous variables between groups. Spearman's correlation analysis was utilized for correlational analysis. By median value, continuous variables were typically divided into high‐ and low‐expression subgroups. For survival analysis, a Kaplan–Meier analysis with log‐rank regression was conducted. For prognostic analysis, Cox proportional hazard models were applied. All data procession was performed on the platform of R software.

## 3 RESULTS

### 3.1 HMOX1 pathway signature associated with unfavorable prognosis under TKI + IO therapy

**FIGURE 1:**
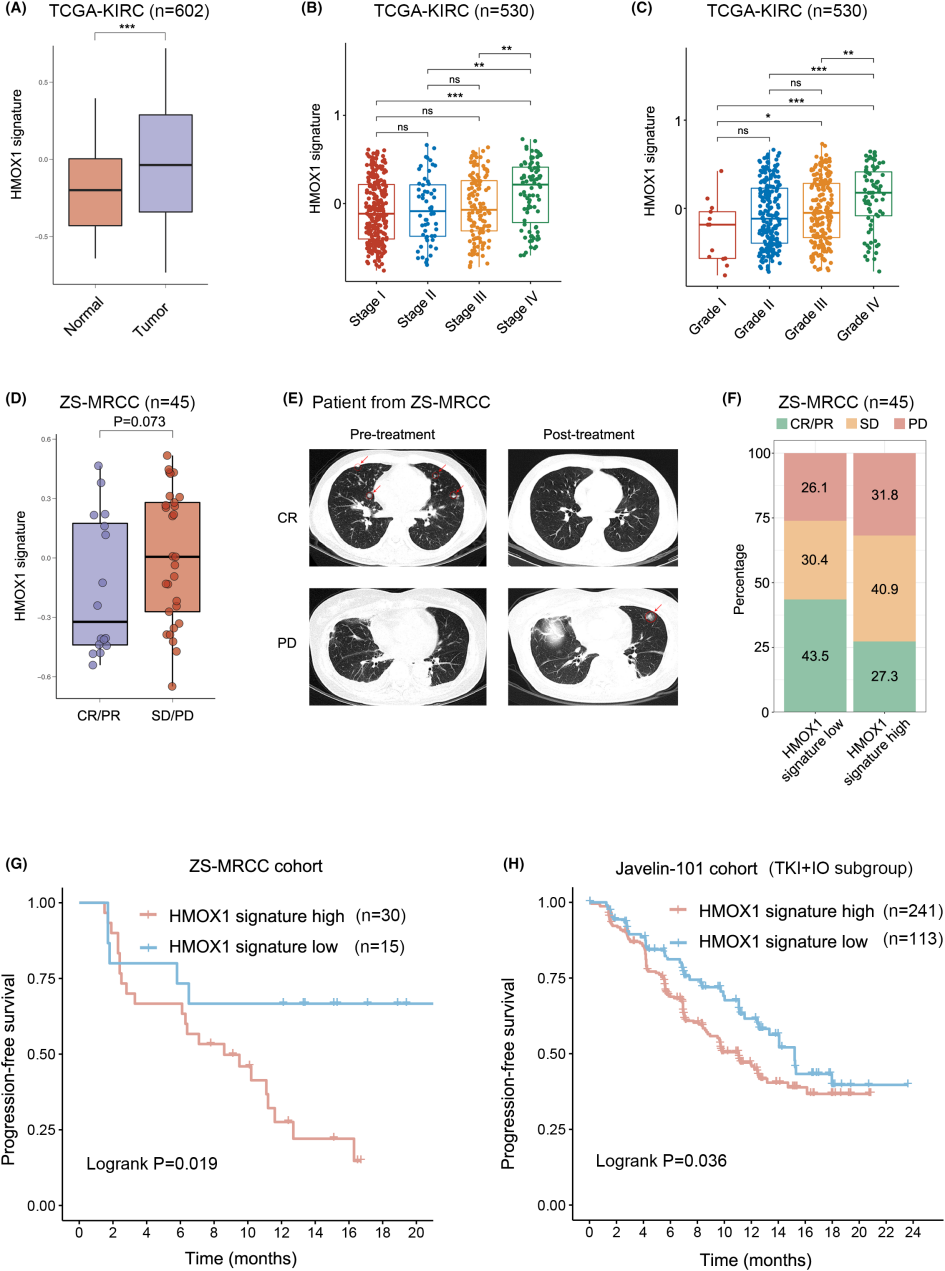
Prognostic value of HMOX1 pathway signature for TKI + IO therapy in advanced renal cell carcinoma (RCC). (A) Expression of HMOX1 pathway signature in RCC and normal tissues. *p* values, Kruskal–Wallis H test. (B, C) Association between HMOX1 pathway signature and TNM stage/ISUO grade in RCC. *p* values, Kruskal–Wallis H test. *, *p* < 0.05; **, *p* < 0.01; ***, *p* < 0.001; ns, not significant. (D) Expression of HMOX1 pathway signature between responders and non‐responders of TKI + IO combination therapy in the ZS‐MRCC cohort. *p* values, Kruskal–Wallis H test. (E) Pre‐ and post‐treatment computed tomography images of PD and CR patients treated TKI + IO combination therapy. (F) Therapeutic response according to HMOX1 pathway signature in ZS‐MRCC cohort under TKI + IO combination therapy. (G, H) PFS after TKI + IO therapy according to HMOX1 signature in the ZS‐MRCC cohort and the Javelin 101 cohort of TKI + IO combination therapy. *p* value, Kaplan–Meier analysis and log‐rank test.

There is an urgent need to establish predictive biomarkers. As mentioned in the introduction section, HMOX1 pathway activation may inhibit antitumor CD8^+^ T‐cell activity in the TME. We performed GSVA to build a signature for HMOX1 pathway in the TCGA‐KIRC cohort. Expression of HMOX1 pathway signature was elevated in RCC tissues compared with non‐tumor tissues (Figure 1A) (*p* < 0.001). In addition, HMOX1 pathway signature was associated with advanced TNM stage and ISUP grade in RCC of TCGA‐KIRC cohort (Figure 1B,C). A range of therapeutic benefits was demonstrated in our ZS‐MRCC cohort (Figure 1D–F). Therefore, we would like to investigate whether HMOX1 signature levels vary between responders (CR/PR) and non‐responders (SD/PD). Both univariate and multivariate Cox regression analysis was performed. Clinical and pathological parameters, including histology, grade, IMDC group, along with HMOX1 pathway signature were incorporated into the Cox regression model. We found that HMOX1 signature was a prognostic factor, which was independent of the above clinical and pathological parameters based on PFS (univariate: hazard ratio [HR] = 3.067, 95% confidence interval [CI] = 1.143–8.233, *p* = 0.026; multivariate: HR = 3.164, 95% CI = 1.135–8.821, *p* = 0.028; Table S4). In ZS‐MRCC cohort, HMOX1 signature was elevated in non‐responders to TKI + IO therapy, compared with responders. Probably because of the limited cohort size, the *p*‐value is close to but not statistically significant (Figure 1D). Patients with low HMOX1 signature had a higher response rate (PR/CR: 43.5% vs. 27.3%; PD: 26.1% vs. 31.8%; Figure 1F) and longer PFS (*p* = 0.019, Figure 1G) than those with a high HMOX1 signature. To verify the prognostic significance of HMOX1 pathway signature, we performed GSVA with Javelin 101 cohort transcriptome data. In the Javelin 101 cohort, those with low HMOX1 signature had a better prognosis (Figure 1H, *p* = 0.036).

### 3.2 HMOX1 signature associated with the clinical benefit of TKI + IO compared with TKI

**FIGURE 2:**
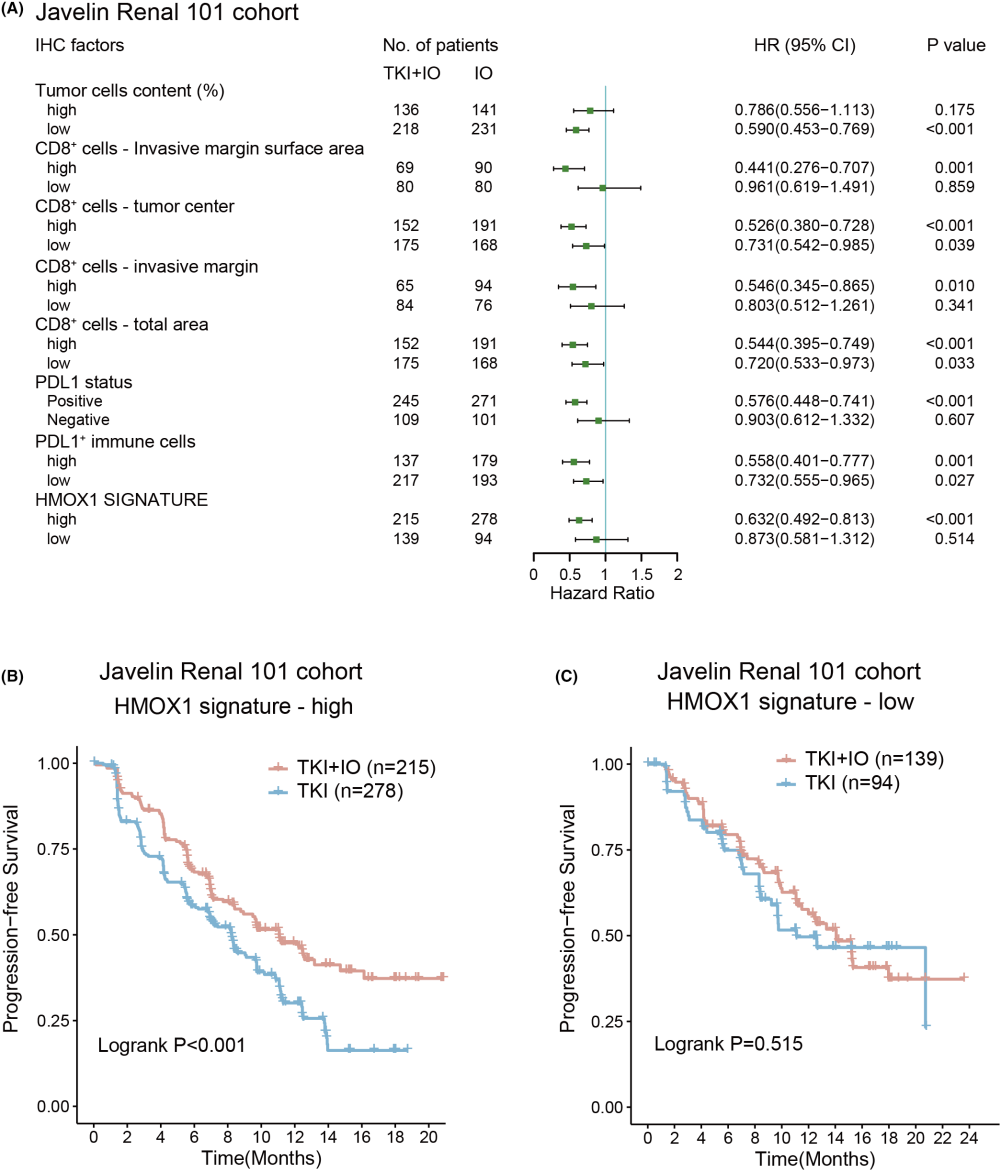
HMOX1 signature associated with clinical benefit of TKI + IO compared with TKI. (A) Different clinical benefit of TKI + IO versus TKI monotherapy for PFS in subgroups defined by IHC markers and expression of HMOX1 pathway signature. HR and *p* values, Cox regression model; (B, C) PFS of TKI + IO or TKI monotherapy in different HMOX1 pathway signature expression subgroups. (B) With high HMOX1 pathway signature, (C) with low HMOX1 pathway signature. *p* value, Kaplan–Meier analysis, and log‐rank test.

The latest EAU Guideline of RCC recommended TKI + IO combinations as standard first‐line therapy, while TKI monotherapy was recommended as alternative therapy as well.^6^ Although clinical studies showed that TKI + IO had a greater clinical benefit than TKI monotherapy, patients under TKI + IO treatment exhibited a variety of therapeutic benefits. IHC markers, including PDL1, CD8‐positive cell total area, and CD8 invasive margin surface area, may predict the prognosis of TKI + IO combination therapy versus TKI monotherapy. Thus, univariate Cox analysis was performed to determine the prediction potential of these markers in the Javelin 101 cohort (Figure 2A). Invasive margin refers to the 1000‐m‐wide interface between malignant and adjacent normal tissue. We found that high HMOX1 signature may indicate that these patients could probably benefit from TKI + IO combination therapy rather than TKI monotherapy, meaning that TKI + IO showed better PFS versus TKI monotherapy only in the high HMOX1 signature arm (*p* < 0.001, Figure 2A). Kaplan–Meier analysis confirmed the result of Cox regression results in HMOX1 signature arm patients (*p* < 0.001, Figure 2B). In contrast, there was no significant difference in PFS between the two therapeutic strategies in the low HMOX1 signature arm (*p* = 0.515, Figure 2C). These findings demonstrated that HMOX1 pathway signature, as well as PDL1, CD8^+^ cells, and tumor cell content may be associated with the prognosis of TKI + IO, as opposed to TKI monotherapy. And only in the high HMOX1 signature arm, high PDL1 or PDL1‐positive arms, higher CD8^+^ cells arm, and higher tumor cell content arms, TKI + IO was more effective than TKI monotherapy.

### 3.3 Interaction between HMOX1 signature and CD8 ^+^ T cells for TKI + IO benefit stratification

**FIGURE 3:**
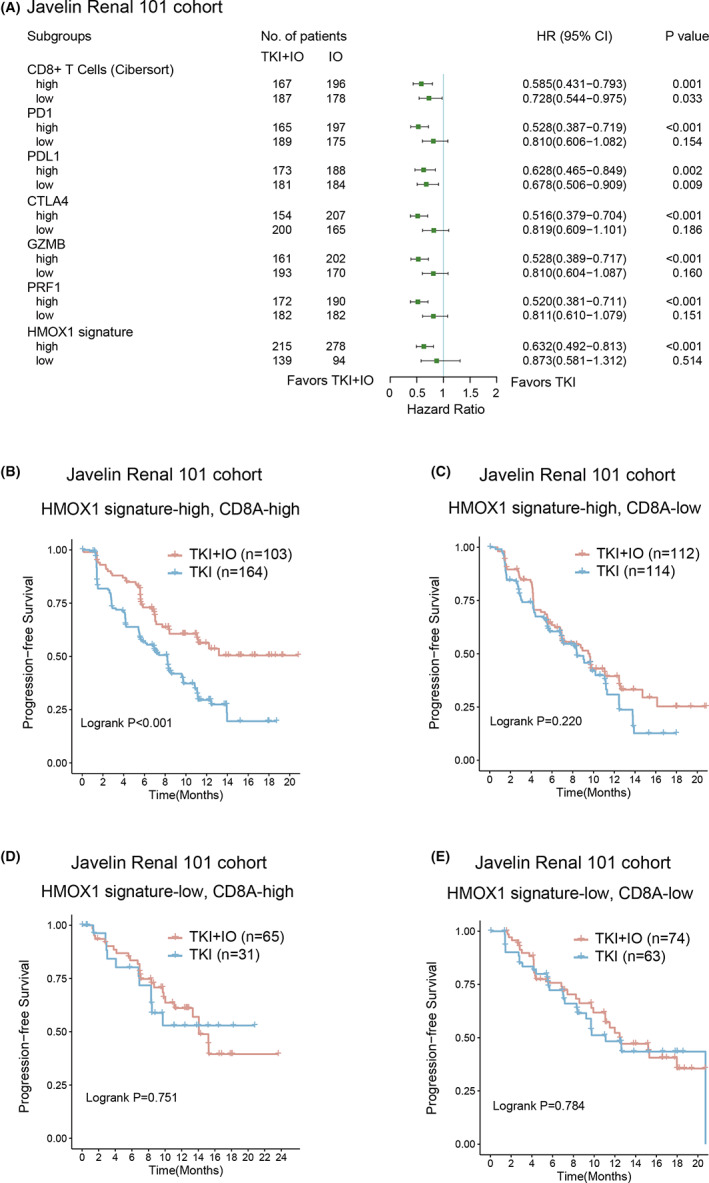
Interaction between HMOX1 signature and CD8+ T cells for TKI + IO benefit stractification. (A) Different clinical benefit of TKI + IO versus TKI monotherapy for PFS in subgroups defined by TME components and expression of HMOX1 pathway signature. HR and *p* values, Cox regression model; (B–E) PFS of TKI + IO in different HMOX1 signature expression and CD8^+^ T‐cell subgroups. (B) With high HMOX1 signature and high CD8A, (C) with high HMOX1 signature and low CD8A, (D) with low HMOX1 pathway and high CD8A, (E) with low HMOX1 signature and low CD8A. *p* value, Kaplan–Meier analysis, and log‐rank test.

IHC markers illustrated that the PDL1 and CD8^+^ cells in different area may serve as a predictor of TKI + IO therapy in the Javelin 101 cohort. Subsequently, we investigated whether other TME components are linked with prognosis in the Javelin 101 cohort. Cibersort analysis was performed.^29^ Then, a univariate Cox analysis was conducted to determine the predictive ability of these markers (Figure 3A). CD8^+^ T cells appeared to be stronger prognostic factor of TKI + IO combination therapy across all 22 cell types of cibersort analysis in the Javelin 101 cohort, meaning that in the subgroup with high CD8^+^ T cells, TKI + IO is more effective than TKI monotherapy, whereas, in the subgroup with low CD8^+^ T cells, there is no difference between TKI + IO and TKI. Based on these results, we hypothesized that TKI + IO would be most beneficial for patients together with more CD8^+^ T cells and a high HMOX1 pathway signature. As a result, a Kaplan–Meier analysis was conducted, which confirmed our hypothesis. In the high HMOX1 subgroup, CD8^+^ T cell was a prognostic indicator, while in the low HMOX1 subgroup, it was not (Figure 3B–E).

### 3.4 Interaction between HMOX1 pathway signature and mutations in advanced RCC

**FIGURE 4:**
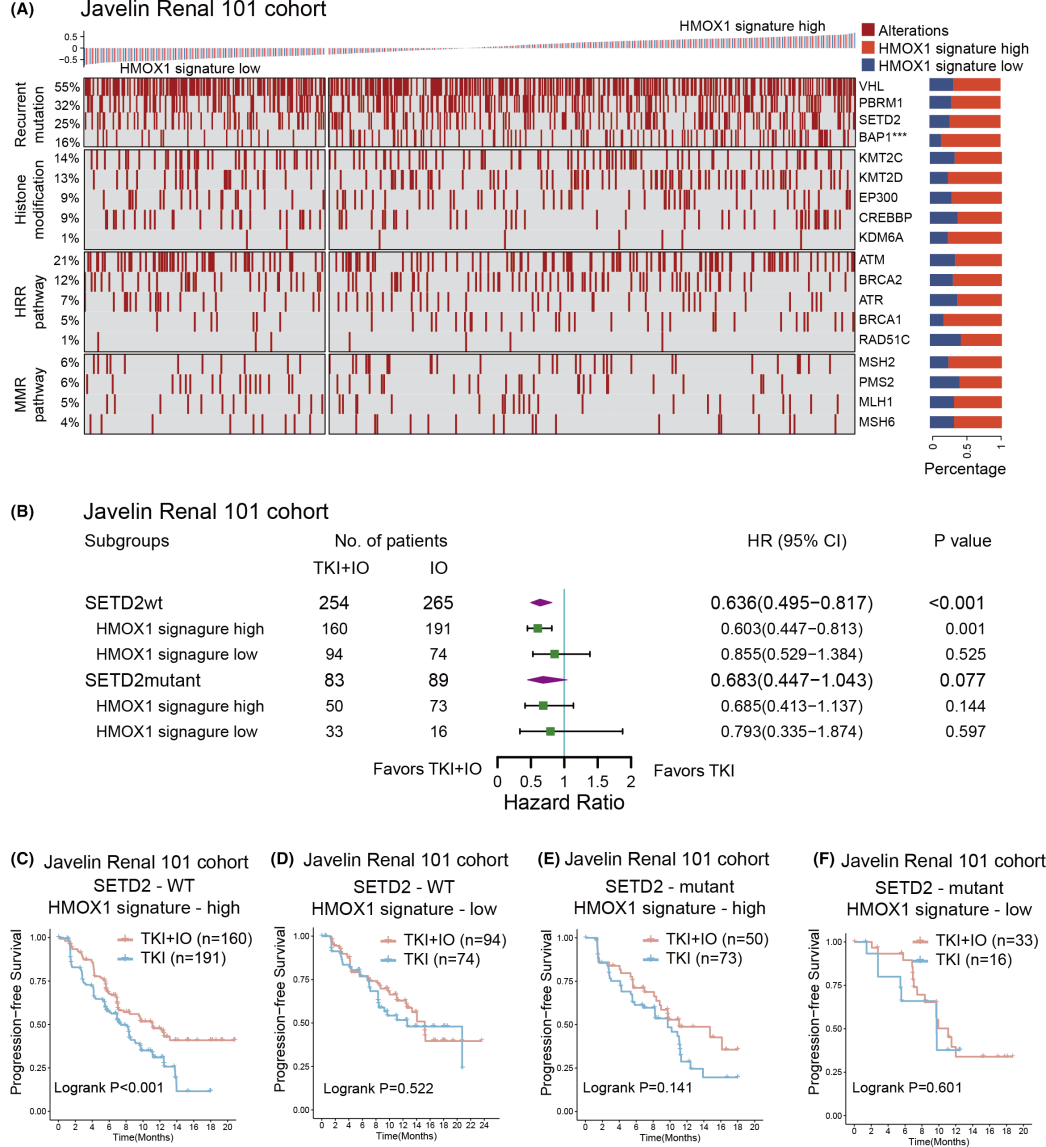
Interaction between HMOX1 pathway signature and mutations in advanced RCC. (A) Waterfall plot showing genomic mutations ranked by HMOX1 signature expression in the JAVELIN‐101 cohort. *p* values, chi‐square test. ***, *p* < 0.001; (B) different clinical benefit of TKI + IO versus TKI monotherapy for PFS in subgroups defined by SETD2 mutational status and HMOX1 signature expression. HR and *p* values, Cox regression model; wt, wild type, mt, mutant. (C–F) PFS of TKI + IO or TKI monotherapy in the HMOX1 signature and SETD2 mutation status subgroups. (C) with high‐HMOX1 signature expression and SETD2 wild type, (D) with low‐HMOX1 signature expression and SETD2 wild type, (E) with high‐HMOX1 signature expression and SETD2 mutant type, (F) with low‐HMOX1 signature expression and SETD2 mutant type. *p* value, Kaplan–Meier analysis and log‐rank test.

Molecular subtyping based on genetic alterations may guide individualized therapy decisions. We established an overview of the genomic mutations and pathway mutations of advanced RCC in the JAVELIN‐101 cohort, ranked by the HMOX1 signature (Figure 4A). Frequent mutations in advanced RCC were observed in the JAVELIN‐101 cohort, including VHL (55%), PBRM1 (32%), and SETD2 (25%). Only BAP1 mutation showed a statistically significant link with HMOX1 signature levels (*p* < 0.001, Figure 4A).

SETD2 was demonstrated as one of the most frequently mutated genes in RCC.^30^ The methylation of STAT1 is essential for the interferon‐dependent immune response, and it has been reported that SETD2 mediates this process.^31^ Furthermore, SETD2 mutation was associated with the immunotherapy response rate in multiple tumor types.^32^ In the JAVELIN‐101 cohort, TKI + IO improved PFS compared to TKI monotherapy only in the SETD2‐wild type (SETD2‐wt) subgroup (HR 0.636, 95% CI 0.495–0.817, *p* < 0.001, Figure 4B). Among the SETD2‐wt subgroup, only in the high HMOX1 signature arm, TKI + IO was more effective than TKI (HR 0.603, 95% CI 0.447–0.813, Figure 4B), while in the SETD2‐mutation subgroup, TKI + IO was unable to show any advantage (HR 0.683, 95% CI 0.447–1.043, Figure 4B). The phenomenon was also validated by Kaplan–Meier analysis in the JAVELIN‐101 cohort (SETD2‐wt & high‐HMOX1, *p* < 0.001; SETD2‐wt & low‐HMOX1, *p* = 0.522; SETD2‐mutant & high‐HMOX1, *p* = 0.141; SETD2‐mutant & low‐HMOX1, *p* = 0.601, Figure 4C–F). The results revealed the integration of the HMOX1 pathway signature and SETD2 mutation status may potentially guide the therapeutic decision‐making of RCC.

### 3.5 Infiltration and dysfunction of T cells in high‐HMOX1 signature RCC

**FIGURE 5:**
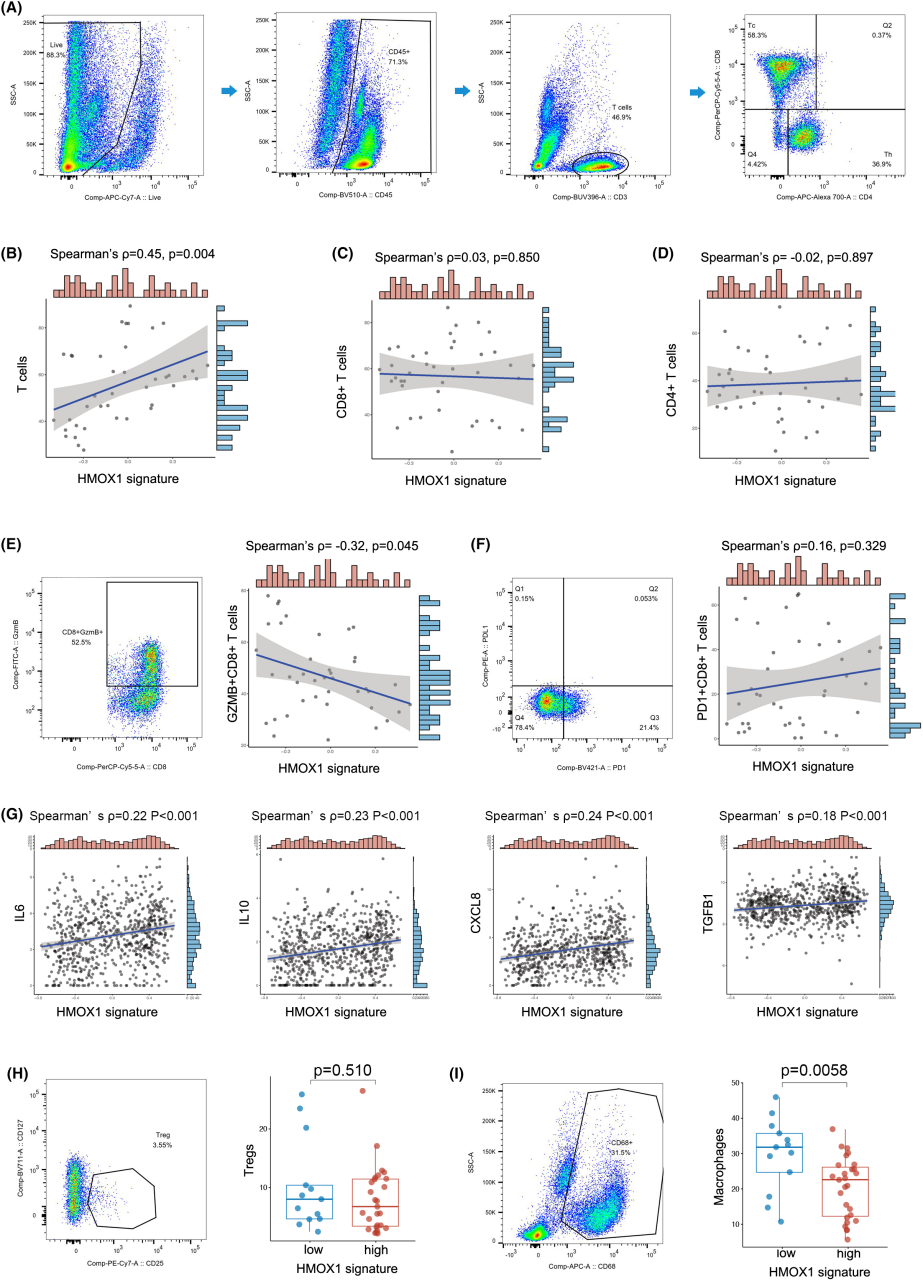
Infiltration and dysfunction of T cells in high‐HMOX1 signature RCC. (A) Gating strategies of T cells, CD8^+^ T cells and CD4^+^ T cells by flow cytometry of fresh tumor samples in the ZS‐HRRCC cohort. (B–D) Association between HMOX1 signature and T cells (B), CD8^+^ T cells (C) and CD4^+^ T cells (D) in the ZS‐HRRCC cohort. ρ and *p* values, Spearman's rank‐order correlation. (E, F) Gating strategy of GZMB^+^CD8^+^ T cells (E), PD1^+^CD8^+^ T cells (F), and their association with HMOX1 signature in ZS‐HRRCC cohort by flow cytometry. ρ and *p* values, Spearman's rank‐order correlation. (G) Association between IL‐6/IL‐10/CXCL8/TGF‐β1 expression and HMOX1 signature in the Javelin cohort. ρ and *p* values, Spearman's rank‐order correlation. Gating strategy of Tregs (H) and macrophages (I), and their association with HMOX1 signature. ρ and *p* values, Spearman's rank‐order correlation.

HMOX1 pathway signature was revealed to be related with TKI + IO therapy efficacy. We would like to investigate the correlation between HMOX1 pathway signature and immune cells. Thus, we applied flow cytometry to freshly‐resected RCC samples in our ZS‐HRRCC cohort (Figure 5A). HMOX1 signature was positively associated with TILs (Spearman's ρ = 0.45, *p* = 0.004, Figure 5B). However, neither CD8^+^ T cells (Spearman's ρ = 0.03, *p* = 0.850, Figure 5C) nor CD4^+^ T cells (Spearman's ρ = −0.02, *p* = 0.897, Figure 5D) correlated significantly with HMOX1 signature. CD8 and CD4 immunohistochemistry confirmed the flow cytometry results (data not shown).

Clearly, HMOX1 was unable to affect the number of CD8^+^ cells and CD4^+^ cells, resulting in the effectiveness of TKI + IO therapy. Thus, we conducted further discovery on whether T‐cell dysfunction occurred in samples with a high HMOX1 signature. T‐cell exhaustion is responsible for tumor cells' immune tolerance in TME. Exhausted T cells express a lower level of cytotoxic factors and a higher level of inhibitory receptors. We measured the expression of GZMB and PD1 on CD8^+^ and CD4^+^ T cells to assess their cytotoxic capacity (Figure 5E,F). A negative correlation was found between GZMB^+^ CD8^+^ T cells and HMOX1 signature (Spearman's ρ = −0.32, *p* = 0.045, Figure 5E). In contrast, PD1^+^ CD8^+^ T cells was not correlated with HMOX1 (Spearman's ρ = 0.16, *p* = 0.329, Figure 5F). Regarding to CD4^+^ T cells, neither GZMB^+^ CD4^+^ T cells nor PD1^+^ CD4^+^ T cells showed significant correlation with HMOX1 signature (data not shown). Interestingly, macrophages were significantly increased in low HMOX1 subgroup (*p* = 0.0058, Figure 5I), while Tregs did not differ in high‐ and low‐ subgroups (*p* = 0.510, Figure 5H).

As dysfunctional T cells may be a result of increasing immunosuppressive cytokines, we performed spearman association analysis between HMOX1 signature and IL‐6, IL‐10, CXCL8, and TGF‐β1. Positive correlation was found between HMOX1 signature and IL‐6 (Spearman's ρ = 0.22, *p* < 0.001), IL‐10 (Spearman's ρ = 0.23, *p* < 0.001), CXCL8 (Spearman's ρ = 0.24, *p* < 0.001), TGF‐β1 (Spearman's ρ = 0.18, *p* < 0.001) (Figure 5G).

### 3.6 An integrated risk score for TKI + IO benefit prediction

**FIGURE 6:**
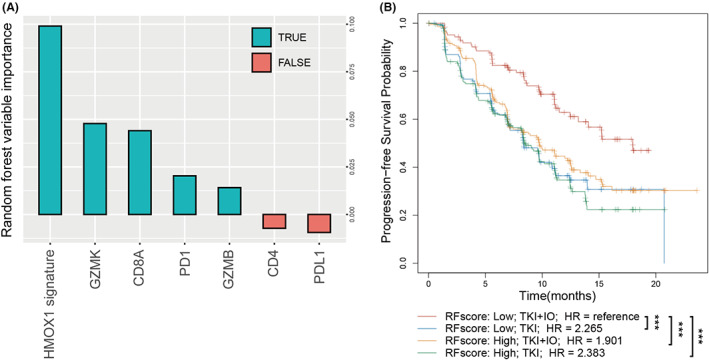
An integrated risk score for TKI + IO benefit prediction. (A) Random forest strategy for risk score construction, involving HMOX1 signature, PD1, PDL1, CD8A, CD4, GZMB, GZMK. (B) Kaplan–Meier analysis of advanced RCC according to risk score and therapeutic regimens. *p* value, Kaplan–Meier analysis and log‐rank test.

According to the PFS of the Javelin 101 study, TKI + IO appears to be a better treatment option for patients with mRCC. However, the therapeutic benefits of TKI + IO varied from individual to individual. There always was a subgroup that did not respond well to TKI + IO therapy. There is an urgent need to develop a model capable of identifying the subgroup that reacts best to TKI + IO therapy. HMOX1 pathway signature showed a potential predictive and prognostic value for TKI + IO combination therapy. Thus, we would like to build a model based on it. Random forest, one of the most popular machine learning techniques, was implemented. The HMOX1 signature, PD1, PDL1, CD8A, CD4, GZMB, and GZMK were enrolled as the parameters for model construction. The contribution of each parameter to the final model was then examined. The HMOX1 signature contributed the most to the random forest model, as expected (Figure 6A). Subsequently, the prognostic value of our model (RFscore) was verified by Kaplan–Meier analysis. The results demonstrate that patients with low RFscore in our final model have the best PFS to TKI + IO combination therapy (Figure 6B).

## 4 DISCUSSION

HO‐1, encoded by HMOX1, is the enzyme that catalyzes the conversion of free heme into three major biologically active by‐products: carbon monoxide, ferrous iron, and biliverdin (which is then converted to bilirubin). In various pathological contexts, HO‐1 has been shown to have important cytoprotective, anti‐inflammatory, antioxidant, and anti‐apoptotic properties.^33^, ^34^, ^35^, ^36^ A growing body of evidence also suggests that HO‐1 may promote tumor development. HMOX1 is expressed in many types of solid cancer and is often associated with a poor prognosis.^37^, ^38^ In the current study, we identified an increase in HMOX1 pathway signature expression in RCC tissues (Figure 1A), and was related with stage and grade (Figure 1B,C).

The HMOX1 pathway plays an important and intricate function in the TME. Previous research demonstrated that HO‐1 is strongly expressed in monocytic cells in the TME once they develop into tumor‐associated macrophages. Knockout of HMOX1 gene in myeloid cells restored CD8^+^ T‐cell proliferation and function, resulting in the therapeutic anticancer vaccine's positive effects.^39^ In accordance with this result, it has been demonstrated that inhibiting HO‐1 promotes cytotoxic antitumor immune response.^13^, ^40^ These findings indicated HO‐1 as a promising therapeutic target for reforming the TME and enhancing the antitumor immune response. Immune contexture, which is characterized by the density, composition, functional status, and structure of the immune cell infiltrate, is a significant determinant of tumor growth and therapeutic response. In our ZS‐HRCC cohort, using FACS, we examined the immunological context in the present work. Interestingly, despite the fact that the number of tumor‐infiltrating T cells was increased in high HMOX1 signature samples (Spearman's ρ = 0.45, *p* = 0.004, Figure 5B), CD8^+^ T cells, CD4^+^ T cells, Tregs, and macrophages were not associated with HMOX1 signature. However, the function of CD8^+^ T cells was disrupted in samples with elevated levels of HMOX1 signature, as indicated by a decrease in GZMB^+^ CD8^+^ T cells (Spearman's ρ = −0.32, *p* = 0.045, Figure 5E). Dysfunctional CD8^+^ T cells, which is a terminally exhausted phenotype, have been observed in ccRCC and were more abundant in advanced disease.^41^, ^42^, ^43^, ^44^

In renal cancer, the well‐known tyrosine kinase receptor c‐Met is up‐regulated, contributing to tumor growth and patient survival. Previous research demonstrated that c‐Met‐mediated signaling activated the Ras signaling pathway and inhibits cellular apoptosis by overexpressing the cytoprotective protein HO‐1. Furthermore, HO‐1‐dependent c‐Met signaling regulated the production of the PD‐L1 on renal cancer cells, hence preventing immune escape of tumor cell. When either Ras or HO‐1 was inhibited, c‐Met‐mediated signaling was unable to produce PD‐L1 at the optimal level.^45^ In current study, we proved that both HMOX1 signature level and PD‐L1 expression status were prognostic factors for TKI + IO combination therapy. Patients with low HMOX1 signature had a higher response rate (PR/CR: 43.5% vs. 27.3%; PD: 26.1% vs. 31.8%; Figure 1F) and longer PFS (*p* = 0.019, Figure 1G; *p* = 0.036, Figure 1H) under the treatment of TKI + IO therapy.

Interestingly, IL‐6 and IL‐10, poor prognostic factors, were positively associated with HMOX1 pathway (Figure 5G). Although IL‐6 is incapable of directly inducing the activation and cytokine production of CD8^+^ T cells, CD8^+^ T cells were not efficiently primed and activated the anti‐tumor function because of the insufficient helper activity of IL‐6‐sensitized CD4^+^ T cells, resulting in tumor development.^46^ IL‐10 was also demonstrated to directly decrease the antigen sensitivity of CD8^+^ T cells and restrict CD8^+^ T‐cell activation and function through modification of cell surface glycosylation.^47^ In addition, suppressive cytokines of CXCL8 and TGF‐β1 were positively related to HMOX1 pathway expression.^48^, ^49^ These findings indicated that HMOX1 may play a crucial role in T‐cell dysfunction, which may lead to immunosuppression and treatment resistance in RCC.

The latest EAU Guideline of RCC recommends TKI + IO combinations as standard first‐line therapy, and TKI monotherapy is recommended as alternative therapy as well.^6^ Multiple clinical studies showed that TKI + IO had a longer PFS than TKI alone. However, patients under TKI + IO treatment exhibited a variety of therapeutic benefits. No clinically appropriate biomarker exists for clinical diagnosis. Thus, we also performed random forest to construct a model by the HMOX1 signature, PD1, PDL1, CD8A, CD4, GZMB, GZMK. The HMOX1 signature contributed the most to the random forest model (Figure 6A) and the model was verified by Kaplan–Meier analysis (Figure 6B). This phenomenon indicated that the quantity and function of HMOX1 signature play a critical role in IO+TKI sensitivity.

This study has several limitations. The retrospective approach and limited sample sizes might result in bias. Further prospective validation in larger cohorts is expected. Additionally, the mechanism of the relationship between HMOX1 pathway and T‐cell exhaustion would be discovered in the future.

## AUTHOR CONTRIBUTIONS

**Xianglai Xu:** Investigation (lead); writing – review and editing (lead). **Sihong Zhang:** Data curation (equal); writing – original draft (equal). **Ying Wang:** Investigation (equal). **Yanjun Zhu:** Supervision (equal). **Jiajun Wang:** Investigation (equal); supervision (equal); writing – review and editing (equal). **Jianming Guo:** Supervision (lead); writing – review and editing (equal).

## FUNDING INFORMATION

This study was funded by grants from the National Natural Science Foundation of China (81700660, 81902898, 81772696, 81974393), Shanghai Sailing Program (19YF1407900), and Experimental Animal Project of Shanghai Science and Technology Commission (19140905200). All these study sponsors have no roles in the study design, in the collection, analysis, and in the interpretation of data.

## CONFLICT OF INTEREST STATEMENT

The authors declare that they have no competing interests.

## ETHICS STATEMENT

The study followed the Declaration of Helsinki and was approved by the Clinical Research Ethics Committee of Zhongshan Hospital, Fudan University (B2021‐119). Informed consent was obtained from each participant.

## Supporting information


Table S1.

Table S2.

Table S3.

Table S4.

## Data Availability

The datasets in the current study are open to the public at the TCGA (https://xena.ucsc.edu/) and Javelin 101 clinical trial (https://www.nature.com/articles/s41591‐020‐1044‐8). Further inquiries can be directed to the corresponding authors. The data of our cohorts that support the results of this study are available from the corresponding author upon reasonable request.
