# Supplementary material for: HMOX1 pathway signature predicts clinical benefit from immunotherapy plus tyrosine kinase inhibitor therapy in advanced renal cell carcinoma
Source: Cancer Med. 2023 Apr 9;12(9):10512–25. doi: 10.1002/cam4.5787 (PMC10225196; doi:10.1002/cam4.5787)
Supplement: Supplementary file 1 — Table S1. [file CAM4-12-10512-s004.doc]

| Table S1. Inclusion and exclusion criteria for ZS-MRCC and ZS-HRRCC cohorts. | | |
| --- | --- | --- |
| Cohorts | Inclusion Criteria | Exclusion Criteria |
| **ZS-MRCC (n=45)** | Metastatic RCC | Refusal to participate |
|  | Combined TKI+IO therapy | Unavailable sample |
|  | No history of other malignancy | Not passing sample quality control |
|  | Available tumor sample | Loss of follow-up |
| **ZS-HRRCC (n=40)** | Localized or locally-advanced RCC | Refusal to participate |
|  | Radical nephrectomy | Not passing sample quality control |
|  | Stage and Grade:   - pT2a, Grade 3 or 4, N0, M0 - pT2b, any Grade, N0, M0 - pT3/T4, any Grade, N0, M0 - any T Stage, any Grade, N1, M0 | Unavailable tumor sample |
|  | No neo-adjuvant therapy |  |
|  | Available tumor sample |  |
