# Supplementary material for: HMOX1 pathway signature predicts clinical benefit from immunotherapy plus tyrosine kinase inhibitor therapy in advanced renal cell carcinoma
Source: Cancer Med. 2023 Apr 9;12(9):10512–25. doi: 10.1002/cam4.5787 (PMC10225196; doi:10.1002/cam4.5787)
Supplement: Supplementary file 2 — Table S2. [file CAM4-12-10512-s002.doc]

| Table S2. Baseline demographic and clinical characteristics of the ZS-MRCC cohort. | |
| --- | --- |
|  | ZS-MRCC cohort, n=45 |
| Age, median (range) | 62 (18-79) |
| Gender |  |
| Male | 25 (55.6%) |
| Female | 20 (44.4%) |
| Nephrectomy | 45 (100.0%) |
| Histology |  |
| Clear cell | 31 (68.9%) |
| None clear cell | 14 (31.1%) |
| ISUP grade |  |
| G2 | 22 (48.9%) |
| G3 | 15 (33.3%) |
| G4 | 6 (13.3%) |
| Regimens |  |
| Axitinib/Tislelizumab | 22 (48.9%) |
| Axitinib/Sintilimab | 8 (17.8%) |
| Lenvatinib/Pembrolizumab | 15 (33.3) |
| Line of therapy |  |
| First-line | 20 (44.4%) |
| Second-line | 13 (28.9%) |
| Third-line | 12 (26.7%) |
| IMDC risk groups |  |
| Favorable | 8 (17.8%) |
| Intermediate | 31 (68.9%) |
| Poor | 6 (13.3%) |
| Best response |  |
| CR | 2 (4.4%) |
| PR | 14 (31.1%) |
| SD | 16 (35.6%) |
| PD | 13 (28.9%) |
| HMOX1 signature |  |
| Low | 15 (33.3%) |
| High | 30 (66.7%) |
|  | |
