# Supplementary material for: HMOX1 pathway signature predicts clinical benefit from immunotherapy plus tyrosine kinase inhibitor therapy in advanced renal cell carcinoma
Source: Cancer Med. 2023 Apr 9;12(9):10512–25. doi: 10.1002/cam4.5787 (PMC10225196; doi:10.1002/cam4.5787)
Supplement: Supplementary file 3 — Table S3. [file CAM4-12-10512-s003.doc]

| Table S3. |  | | |
| --- | --- | --- | --- |
| Signature | Gene | | |
| **HMOX1** | BACH1 | CSNK2A1 | CSNK2A2 |
|  | CSNK2B | CUL1 | CUL3 |
|  | FBXL17 | HM13 | HMOX1 |
|  | KEAP1 | MAFK | NFE2L2 |
|  | PRKCD | PSMA1 | PSMA2 |
|  | PSMA3 | PSMA4 | PSMA5 |
|  | PSMA6 | PSMA7 | PSMA8 |
|  | PSMB1 | PSMB10 | PSMB11 |
|  | PSMB2 | PSMB3 | PSMB4 |
|  | PSMB5 | PSMB6 | PSMB7 |
|  | PSMB8 | PSMB9 | PSMC1 |
|  | PSMC2 | PSMC3 | PSMC4 |
|  | PSMC5 | PSMC6 | PSMD1 |
|  | PSMD10 | PSMD11 | PSMD12 |
|  | PSMD13 | PSMD14 | PSMD2 |
|  | PSMD3 | PSMD4 | PSMD5 |
|  | PSMD6 | PSMD7 | PSMD8 |
|  | PSMD9 | PSME1 | PSME2 |
|  | PSME3 | PSME4 | PSMF1 |
|  | RBX1 | RPS27A | SEM1 |
|  | SKP1 | SKP2 | UBA52 |
|  | UBB | UBC |  |
