# Supplementary material for: HMOX1 pathway signature predicts clinical benefit from immunotherapy plus tyrosine kinase inhibitor therapy in advanced renal cell carcinoma
Source: Cancer Med. 2023 Apr 9;12(9):10512–25. doi: 10.1002/cam4.5787 (PMC10225196; doi:10.1002/cam4.5787)
Supplement: Supplementary file 4 — Table S4. [file CAM4-12-10512-s001.doc]

| Table S4. Univariate and multivariate cox regression for ZS-MRCC (n=45). | | | | | | | | | |
| --- | --- | --- | --- | --- | --- | --- | --- | --- | --- |
|  | Univariate cox regression | | | |  | Multivariate cox regression | | | |
|  | P-value | Hazard ratio | 95% CI | |  | P-value | Hazard ratio | 95% CI | |
| **Histology** |  |  |  |  |  |  |  |  |  |
| Noncc vs cc | 0.725 | 0.861 | 0.375 | 1.978 |  | 0.551 | 0.759 | 0.306 | 1.879 |
| **Grade** | 0.303 |  |  |  |  | 0.128 |  |  |  |
| G3 vs G2 | 0.274 | 0.613 | 0.256 | 1.472 |  | 0.238 | 0.58 | 0.235 | 1.434 |
| G4 vs G2 | 0.483 | 1.444 | 0.518 | 4.026 |  | 0.192 | 2.144 | 0.681 | 6.743 |
| **IMDC** |  |  |  |  |  |  |  |  |  |
| Intermediate and poor vs favorable | 0.416 | 1.557 | 0.535 | 4.531 |  | 0.548 | 1.418 | 0.454 | 4.43 |
| **HMOX1 signature** |  |  |  |  |  |  |  |  |  |
| High vs low | 0.026 | 3.067 | 1.143 | 8.233 |  | 0.028 | 3.164 | 1.135 | 8.821 |
